# Volatile Emissions from Compressed Tissue

**DOI:** 10.1371/journal.pone.0069271

**Published:** 2013-07-09

**Authors:** Francesca Dini, Rosamaria Capuano, Tillan Strand, Anna-Christina Ek, Margareta Lindgren, Roberto Paolesse, Corrado Di Natale, Ingemar Lundström

**Affiliations:** 1 Department of Electronic Engineering, University of Rome Tor Vergata, Rome, Italy; 2 Department of Medical and Health Sciences, Linköping University, Linköping, Sweden; 3 Department of Chemical Science and Technology, University of Rome Tor Vergata, Rome, Italy; 4 Department of Physics, Chemistry and Biology, Linköping University, Linköping, Sweden; Tecnologico de Monterrey, Mexico

## Abstract

Since almost every fifth patient treated in hospital care develops pressure ulcers, early identification of risk is important. A non-invasive method for the elucidation of endogenous biomarkers related to pressure ulcers could be an excellent tool for this purpose. We therefore found it of interest to determine if there is a difference in the emissions of volatiles from compressed and uncompressed tissue. The ultimate goal is to find a non-invasive method to obtain an early warning for the risk of developing pressure ulcers for bed-ridden persons. Chemical analysis of the emissions, collected in compresses, was made with gas-chromatography – mass spectrometry and with a chemical sensor array, the so called electronic nose. It was found that the emissions from healthy and hospitalized persons differed significantly irrespective of the site. Within each group there was a clear difference between the compressed and uncompressed site. Peaks that could be certainly deemed as markers of the compression were, however, not identified. Nonetheless, different compounds connected to the application of local mechanical pressure were found. The results obtained with GC-MS reveal the complexity of VOC composition, thus an array of non-selective chemical sensors seems to be a suitable choice for the analysis of skin emission from compressed tissues; it may represent a practical instrument for bed side diagnostics. Results show that the adopted electronic noses are likely sensitive to the total amount of the emission rather than to its composition. The development of a gas sensor-based device requires then the design of sensor receptors adequate to detect the VOCs bouquet typical of pressure. This preliminary experiment evidences the necessity of studies where each given person is followed for a long time in a ward in order to detect the insurgence of specific VOCs pattern changes signalling the occurrence of ulcers.

## Introduction

Pressure ulcers are defined as localized injuries to the skin and/or underlying tissue usually located over bony prominences [1]. The primary causes of pressure ulcers are pressure and/or shearing forces, which may result in deformation and ischemia affecting tissue viability [2]. An additional primary factor is accumulated heat in the supporting surface increasing the need for blood supply and the risk of ischemia in the tissue [3], [4]. Pressure ulcers, visible early in the skin, are categorized from I to IV. Category I is described as non-blanchable erythema, category II partial tissue damage, category III full thickness skin loss, and category IV full thickness tissue loss [1].

Since patients in all kinds of care settings and almost every fifth patient treated in hospital care develops pressure ulcers early identification of risk is important [5], [6]. Once category I damage is established, the probability of progress is there if not adequate steps are taken [7]. Studies have shown that individuals living with pressure ulcers suffer a lot of pain and also have a low quality of life [8], [9]. The problem is also united with extensive costs for the society.

There have been many studies on skin emissions and body odours in the past. The connection to certain diseases and disorders has been known for a long time, see for example a recent review by Shirasu and Touhara [10]. One thorough study was published by Gallagher et al. [11], where the profile of volatile organic compounds (VOCs) emitted from the upper back and forearm of healthy persons were examined by GC-MS using two types of extraction techniques, solid-phase micro-extraction (SPME) and solvent extraction, respectively. Their conclusion was that the two body locations share a considerable amount of compounds of both exogenous and endogenous origin, but that still differences could be observed. They also suggested that their investigation could be used as the starting point for a database for both healthy and malignant skin volatile compounds.

It is known that prolonged compression prompts ischemia that implies a smaller oxygen availability in compressed tissue [3], [4], [12], [13], [14]. This leads to a change of the normal metabolism with consequences on the amount and composition of excreted metabolites. Eventually these changes influence the profile of the volatiles emitted by the skin. We have, however, not found any literature data on emissions caused only by mechanical load. A non-invasive method for the elucidation of endogenous biomarkers related to pressure ulcers could be extremely useful if it was able to detect the onset of tissue damage long before a pressure ulcer occurs. This was the background of the present study, which was designed to indicate if there is a difference in general between the volatiles collected on the skin of a compressed and uncompressed tissue, respectively. Bed-ridden patients at a neurological intensive care unit (NIVA) and healthy persons were used as two different types of test groups. The emissions, collected in compresses, were analyzed with GC-MS, i.e. gas chromatography with mass spectrometric identification of the compounds, as well as with an array of chemical sensors and a pattern recognition method (an electronic nose) to demonstrate a possible simple instrumentation for bed-ridden patient screening of pressure ulcers' onset. Such “biomimetic measurement systems” have thus been used to classify emissions in very different cases, ranging from applications in process industry to medical diagnostics [15].

We decided to study healthy and bed-ridden persons without any signs of tissue damage in this first study on skin emissions from compressed tissue. This may seem strange, but since we are aiming for an early warning of tissue damage, knowledge of the expected natural patterns is of utmost importance. One of the main objectives of our pilot study was therefore to find out if there was a difference in skin emission patterns from hospitalized patients (persons confined to beds) and healthy control persons. Secondly we liked to find out if there was a significant difference between the emissions from compressed and non-compressed tissue. The last issue was of course of most interest for the hospitalized patients. The experiments closest to the attempts in this paper are probably those related to the detection of the emissions from melanoma [16], [17].

## Materials and Methods

The research project was approved by the regional ethical review board at the faculty of Health Sciences of Linköping University. Written informed consent was obtained from all patients involved in this study.

### Materials

Gauze used to sample VOCs on the skin was 100% cotton, sterile, 7.5 cm×7.5 cm, in 8 ply compress (SELEFATRADE, Sweden). 20 ml screw top glass vials with PTFE/Silicone septa (Supelco, Bellefonte, PA) were used to hold the compresses after skin sampling. TEGADERM, a transparent plastic bandage (3 M, St. Paul, USA), was used to assure the adhesion of the cotton gauze to skin.

### Method for skin sampling

A total of 18 subjects were analyzed, 9 healthy volunteers and 9 hospitalized patients at the NIVA department of Linköping University Hospital. There were 7 females and 2 males in the group of healthy volunteers and 5 females and 4 males in the patient group. The mean age of the healthy volunteers was 40.9±15.3 and 53.7±15.7 for the patients.

The sterile cotton compress was used to collect the skin volatile compounds. Compresses were applied at the hip region, in correspondence of the bony prominence. The area was washed with water and dried with a compress. A new compress was applied on the skin, it was covered with the same plastic of the compress package, and finally an occluding plastic bandage was applied to guarantee the adhesion of the gauze to skin. This procedure was repeated for both hips, and two samples were collected from each person: one from the compressed site (referred to as site 1) and one from the uncompressed site (site 2). The subject was then asked to rest still on one side for 2 hours. The bandages were removed afterwards (compress on site 2 was removed first to avoid any pressure on this site). The body parts were visually inspected, and the appearance of any redness was documented. Among the healthy subjects, only 2 had redness in the compressed site after the 2 hours of applied pressure. None of the patients got an additional redness after the application of the pressure, and just one of them had a small redness before the application of the compress.

Compresses from both sides were cut into three equal parts and stored in different sealed vials, in order to be analyzed with the GC-MS and the electronic nose, respectively. For each person, a blank compress was also placed into a vial and considered as a reference. All vials were stored at room temperature for 24 h before headspace analysis.

### SPME – GC/MS

Headspace analysis was conducted via solid phase microextraction (SPME) utilizing a 50/30 µm coating of divinylbenzene/Carboxen on a polydimethylsiloxane fiber (DVB/CAR/PDMS), (Supelco, Bellefonte, PA). The fiber type was chosen in accordance with literature suggestion for the extraction of human odor samples [18]. The exposure time of the fiber was 15 hours, which had previously been determined to be the optimal extraction time for collected skin odor samples. All SPME exposures were conducted at room temperature. The samples were analyzed with a GC Hewlett Packard 6890 Series, and a MS Hewlett Packard 5973. The GC was equipped with a Varian Factor Four VF-5MS capillary column, 30 meter long, 0.25 mm diameter, 0.25 µm coating with 5% phenyl, 95% dimethylpolysiloxane low bleed phase. Helium was used as the carrier gas with a flow rate of 1.3 ml/min.

The analytes were desorbed from the fiber at the injection port of the GC with an inlet temperature of 250°C. The GC method began with an initial oven temperature of 40°C for 5 min. The temperature was then ramped at 10°C/min until it reached 300°C, and it was held at 300°C for 2 min (total run time: 33 min.). The mass spectrometer was used with a quadrupole analyzer in electronic ionization mode, scanned over a mass range of *m/z* 30–550 in the full scan mode. The temperature of interface and ion source were kept constant at 280°C. Identification of compounds was performed using the Wiley Registry of Mass Spectral library.

### Statistical analysis

Differences in the composition of volatile compounds were sought between “healthy” and “patients”, to evaluate the difference in skin emission composition that may be related to the bed-ridden condition rather than directly to a given pathology (patients were all staying in the intensive care unit but they were affected by different diseases). Then, we studied whether it was possible to identify within these compounds markers of a prolonged pressure on the tissue, comparing the composition of skin emission in the samples collected from the two sites. The differences were evaluated comparing the area of peaks normalized to the total peak area of each chromatogram using analysis of variance (ANOVA). We considered *p*<0.05 as an acceptable level of confidence.

In addition to the analysis above, we treated GC data in their wholeness using multivariate statistical techniques to explain these different patterns. Indeed, in the Results and Discussion section, we show that the Discriminant Analysis solved by Partial Least Squares (PLS-DA) [19] allows one to discriminate between samples on the basis of peak area.

### Electronic nose

**Figure 1:**
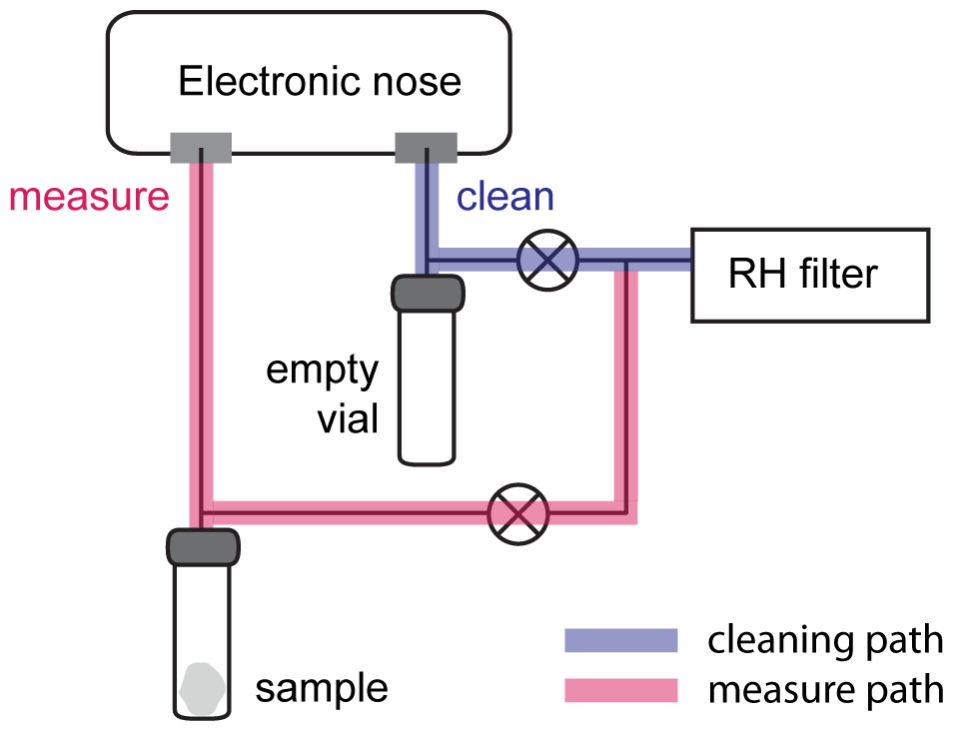
Schematic view of the headspace analysis system with an electronic nose. Two different flow paths are shown, one for the sample measurement phase, that passes though the vial containing the gauze exposed to skin VOCs and one used for flushing the sensors. Both paths pass through a CaCl_2_ cartridge used to filter ambient air and remove the humidity.

The instrument used in our experiments was the last version of the electronic nose developed at the University of Rome Tor Vergata. The e-nose is based on an array of eight quartz microbalances (QMB) coated with different metalloporphyrins [20]. The operating principle of QMBs is based on the variation of mass absorbed onto the quartz coating which causes a proportional change of the resonance frequency. For each sample, the vial headspace was forced to flow at a constant rate of 100 ml/min into the sensor chamber. Figure 1 shows a schematic view of the headspace analysis system. During the measurements, dry air from a CaCl_2_ cartridge was drawn into the vial containing the exposed compress. In the cleaning phase, the sensor chamber was flushed with filtered air passed through an empty vial used to simulate the same flow resistance as for the vial containing the sample. Measurements were performed 15 h after the collection, simultaneously with the end of the SPME exposure. According to the short time delivery strategy [21], the measurement of each sample consists of three short exposures to the vial headspace, whose length was 15 s, 60 s and 30 s, spaced by a cleaning period of 60 s. The response pattern of each sensor is given by the frequency shifts during these three pulses.

## Results and Discussion

### GC-MS data analysis

Our experimental study led to the collection of 54 total ion chromatograms (TIC). For each person, healthy (9) and patient (9), there were three samples, one for the compress itself (blank), one from the compressed side (site 1) and one from the non-compressed side (site 2).

Each TIC was integrated and peaks were matched and aligned in order to obtain a matrix that contains all the peaks found in the whole set of measurements. We disregarded those peaks not above the 1% baseline, as well as those identified as arising from the fiber (siloxanes) and the column. This operation led to the identification of 219 peaks, with varying abundances and occurrences within the samples. Some of them were identified as skin emission as they were not present in the blank compress simultaneously collected during the sampling. We found a number of compounds that in literature are stated as typical of skin emissions, both in those studies sharing our sampling method [22], and in those that made use of sampling methods not involving an adsorber [11]. Among those compounds, there are for instance C_6_–C_12_ aldehydes, 1,2-Ethanediol monoacetate, 1-phenyl-Ethanone, 2,6-Dimethyl 7-Octen-2-ol.

### Comparison of volatile compounds from healthy persons and patients

The differences between patient group and healthy involve a large number of compounds that vary in abundances and occurrences within the two classes. The list reported in Table 1 is a selected subset of compounds that occur in more than 60% of total samples at least in one group and whose ANOVA test returns a *p* value below 0.05. Differences between healthy and patients may be due to the different boundary conditions, as the environment and level of medication.

**Table 1:** Volatile compounds stated as statistically different (p<0.05) in healthy and patients (group) and the effect of the site where samples were collected (site 1 - compressed tissue; site 2 - uncompressed tissue).

		Healthy – site 1			Healthy – site 2			Patients – site 1			Patients – site 2			p	
RT	Compound name	#	mean	std	#	mean	std	#	mean	std	#	mean	std	group	site
7.30	1.2-Ethanediol monoacetate	8	16.3	7.8	8	14.3	10.3	9	7.8	3.9	9	8.4	9.5	0.013	0.84
7.55	Benzene. ethyl-	6	2.7	1.1	6	2.0	0.8	6	0.9	0.7	6	0.8	0.4	0.001	0.35
7.81	Benzene. 1.3-dimethyl-	9	2.1	0.6	9	2.0	1.5	9	0.6	0.5	8	0.6	0.5	0.001	1.00
11.14	Octanal	8	0.9	0.6	8	0.8	0.7	9	0.4	0.2	7	0.4	0.2	0.018	0.99
11.62	Tetramethylsuccinonitrile	8	0.9	0.9	8	0.7	0.8	9	0.3	0.4	7	0.2	0.2	0.021	0.66
13.14	Nonanal	9	3.2	2.6	9	3.2	2.2	9	1.2	0.4	9	1.6	0.7	0.003	0.76
*13.26	Tetradecene	6	0.36	0.1	2	0.21	0.01	4	0.18	0.06	4	0.12	0.04	0.02	0.08
14.76	Dodecane	9	0.5	0.2	9	0.6	0.2	9	0.9	0.4	9	1.1	0.5	0.001	0.47
14.91	Decanal	9	3.0	3.3	9	3.4	3.4	9	1.4	0.5	9	1.7	0.7	0.037	0.71
14.96	Undecane. 2.6-dimethyl-	5	0.4	0.1	6	0.4	0.1	7	0.9	0.1	8	0.9	0.3	0.001	0.88
15.50	Cyclohexane. (1.3-dimethylbutyl)-	7	0.2	0.1	6	0.3	0.1	9	0.5	0.2	9	0.5	0.1	0.001	0.98
15.61	Benzene. 1.3-bis(1.1-dimethylethyl)-	9	0.4	0.2	7	0.5	0.2	8	0.8	0.4	9	0.6	0.3	0.021	0.72
15.78	10-Methylnonadecane	7	0.2	0.1	6	0.2	0.1	8	0.6	0.2	9	0.5	0.2	0.001	0.99
15.89	Octadecane. 2.6-dimethyl-	8	0.3	0.1	7	0.3	0.2	9	1.0	0.5	9	1.1	0.6	0.001	0.66
16.35	Tridecane	9	0.5	0.2	9	0.6	0.3	9	1.5	0.9	9	1.8	0.9	0.001	0.56
16.45	3-methyl-3.4-dihydro-2H-thiopyran	8	0.2	0.1	7	0.3	0.1	7	0.6	0.3	8	0.5	0.2	0.001	0.79
16.59	Heneicosane	5	0.3	0.1	4	0.3	0.1	8	0.7	0.2	8	0.7	0.2	0.001	0.79
*16.93	1.2.3-Propanetriol. triacetate	8	0.5	0.3	8	0.5	0.3	9	1.0	0.3	9	0.7	0.3	0.007	0.19
17.08	Tetracontane. 3.5.24-trimethyl-	8	0.4	0.2	8	0.5	0.2	9	0.7	0.3	9	0.8	0.4	0.002	0.58
17.29	10-Methylnonadecane	7	0.4	0.4	6	0.5	0.4	6	1.5	0.7	7	1.5	0.8	0.001	0.72
17.39	Eicosane	8	0.5	0.2	9	0.6	0.3	9	1.2	0.5	9	1.1	0.5	0.001	0.91
17.71	Propanoic acid. 2-methyl-. 3-hydroxy-2.4.4-trimethylpentyl ester	5	0.3	0.1	6	0.3	0.1	8	0.6	0.2	8	0.7	0.5	0.014	0.98
17.82	Tetradecane	9	1.3	0.5	9	1.5	0.3	9	2.0	0.7	9	2.2	0.6	0.001	0.47
18.83	2.6-di-butyl-2.5-cyclohexadiene-1.4-dione	9	2.1	1.5	9	2.1	1.7	9	0.4	0.3	8	0.4	0.2	0.001	0.91
19.02	Docosane	9	0.9	0.5	9	0.9	0.4	8	0.4	0.2	9	0.4	0.1	0.001	0.82
*19.15	Tritriacontane	6	0.22	0.11	5	0.18	0.08	6	0.15	0.02	5	0.1	0.04	0.01	0.29
*19.20	Pentadecane	9	0.39	0.23	9	0.72	0.4	9	0.26	0.1	9	0.33	0.11	0.01	0.05
19.35	Phenol. 2.6-bis(1.1-dimethylethyl)-4-methyl-	9	0.7	0.3	9	0.9	0.5	8	0.5	0.3	8	0.5	0.2	0.006	0.44
*20.49	1.2-Benzenedicarboxylic acid. diethyl ester	9	0.7	0.2	8	1.1	0.5	9	0.4	0.3	7	0.5	0.2	0.001	0.07
*22.46	Octadecane, 3-ethyl-5-(2-ethylbutyl)-	4	0.1	0.05	4	0.19	0.06	5	0.05	0.02	1	0.08	–	0.01	0.01

The Table lists the retention time, the compound name, the number of occurrence in each case, the average area of peaks normalized to the total peak area and the standard error for each site and each group, and the *p* value of the effect of group. A star in table evidences the compounds that are relevant for the distinction of compressed site.

### Volatile compounds from compressed tissue

Once the discrimination between healthy and hospitalized patients was assessed, our attention focused on seeking compounds that can be related to the mechanical pressure on the tissue. It should be noted that none of the patients developed a pressure ulcer during the study. One patient, however, got a pressure ulcer at another ward one week after our measurement. In general, our data allow the study of compounds that are related to the pressure and to a tissue damage that did not yet lead to the onset of pressure ulcers.

In our dataset, there are compounds which appear in the chromatograms only occasionally, i.e. which are present in only a few samples. We chose not to disregard them because when dealing with biological studies a large degree of variability among samples is always expected. Hence we considered two kinds of analysis, one for recurrent compounds (that appear in more than 50% of samples at least in one group) and one for those compounds that appear only occasionally. The latter can still be connected to the pressure application for particular cases. For recurrent compounds we applied the ANOVA test, and we reported those compounds whose *p* values stay below 0.1, evidenced by a star in Table 1. We found one compound with significantly larger peak area in site 1 in healthy (Tetradecene/13.26), two compounds among patients (1.2.3-Propanetriol. triacetate/16.93 and Tritriacontane/19.15), while three compounds (Pentadecane/19.20, 1.2-Benzenedicarboxylic acid. diethyl ester/20.49 and Octadecane, 3-ethyl-5-(2-ethylbutyl)/22.46) occur with different abundance in the two sites regardless of the condition.

Finally we investigated whether there were occasionally occurring compounds, which appear more often in one site, for healthy, patients, and for the two groups together, with results shown in Table 2.

**Table 2:** Compounds with radically different occurrence in healthy and patients group.

Group	RT	Compound Name	S1	S2
Healthy	11.01	2-Propanol, 1-(2-methoxy-1-methylethoxy)-	2	5
	12.10	2,6,7-trimethyl-Decane	5	2
	16.48	Tridecanol	5	2
	24.66	[(2-fluorophenyl)methyl]-1H-Purin-6-amine	4	1
Patient	17.93	Tritetracontane	8	4
	19.25	Nonahexacontanoic acid	5	2
	21.77	4-(2,6-dimethyl-1-cyclohexen-1-yl) morpholine	5	2
Both	20.59	1-Hentetracontanol	0	7
	23.11	[(2-fluorophenyl)methyl]-1H-Purin-6-amine	5	0
	24.39	5-methoxy-2-methyl-Benzoselenazole	3	0

The table shows the retention time, the name of the compound and the number of occurrences in the two sites for the healthy group, patients or irrespective of group.

Tables 1 and 2 show that it is not possible to identify with certainty one or more volatile compounds, which are emitted from tissue loaded with mechanical pressure. However, it seems that a different pattern of emission may be present due to the compression, even though further studies are needed to identify the markers that represent a warning of pressure ulcers' onset at an early stage.

### Multivariate data analysis

**Figure 2:**
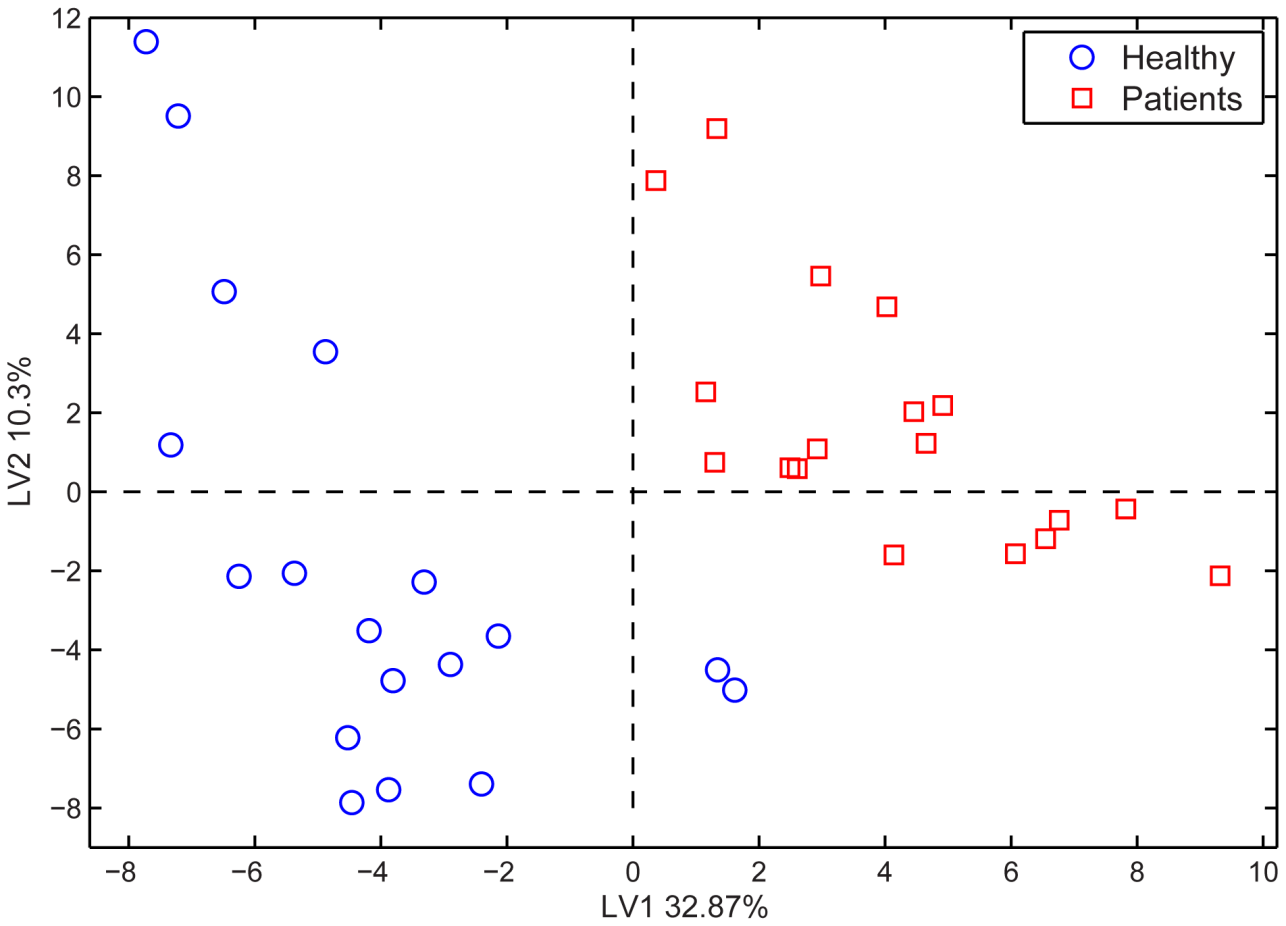
Score plots related to a PLS-DA model built on GC-MS data. The model was calculated using the areas of the peaks listed in Table 1 for healthy subjects and patients. Misclassified samples are H7 site2, P1 site 1 and 2, P7 site 2.

A matrix composed of the areas of relevant peaks was analyzed with PLS-DA. Results were cross-validated by the “leave-one-out” (LOO-) technique that allowed us to choose the correct number of latent variables for our model. The result of the PLS-DA analysis performed on the peaks in Table 1 is shown in Figure 2, where the data, projected onto the first two latent variables, were grouped as patient and healthy, irrespective of site. The results in Figure 2 suggest an overall difference in the emissions from the two groups. Both the first and second latent variables discriminate between groups. Likely the first variable is related to the total amount of the emissions and the second to the composition. Furthermore, about 43% of the variation in the data is explained by these two variables. The LOO validation attributes four samples out of 36 to the wrong category, yielding a classification rate of 88%.

**Figure 3:**
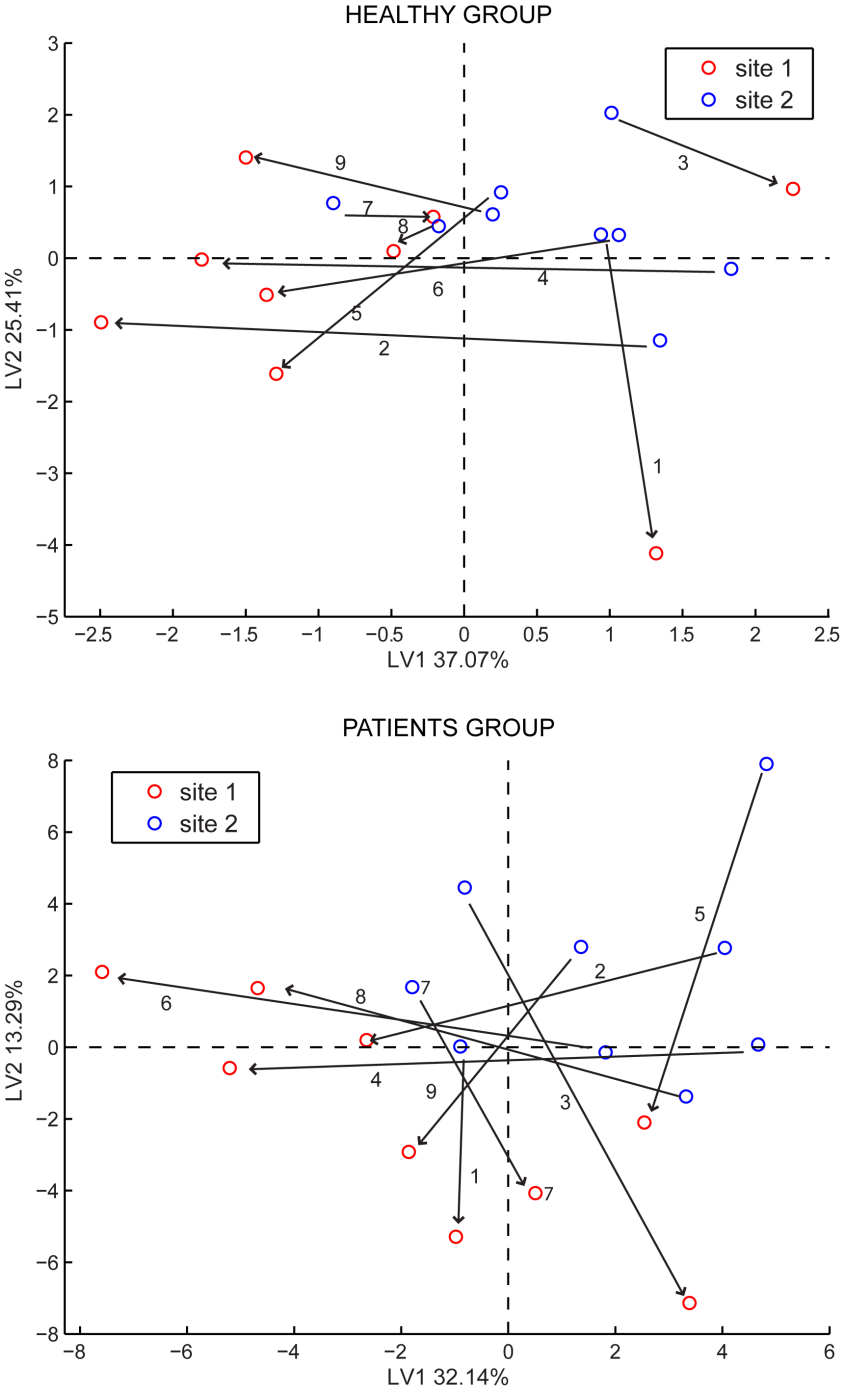
PLS-DA analysis of GC-MS data for healthy subjects and patients separately. The PLS-DA model for the discrimination of compressed and non-compressed site for (A) healthy and (B) patients was calculated using the peaks listed in Tables 2 as discussed in the text. Red points denote the compressed site and blue points the non-compressed site. The arrows connect the points of the two sites for a given person, and indicate the direction of change from uncompressed to compressed tissue sites.

The next issue was to distinguish, independently for each group between the compressed and uncompressed sites. In this case we used a subset of peaks as listed in Tables 1 and 2. In both cases, reported in Figure 3 a–b, a quite evident separation among the two sites is visible. Moreover, it is possible to identify a direction common to almost all samples, which connects the two sites of each person. This direction can be regarded as the trend of change in skin emission composition when the tissue is stressed with mechanical pressure. Our aim will be to identify with next studies, the region of pressure ulcer onset in such a diagram. One of the patients, patient 1 (P1) was diagnosed with a pressure ulcer (category I) only one week after our study. The patient was hospitalized in another ward than NIVA, and was not followed or observed by us. Some interesting observations can be made about the chromatograms of this patient. Most of the compounds were found with similar abundances as for the other patients but one, already listed in Table 2 with retention time 23.11 and identified as [(2-fluorophenyl) methyl]-1H-Purin-6-amine. Although it is one of the smallest in the chromatogram, it occurs almost exclusively in the compressed site of both patients and healthy persons.

### Electronic nose measurements

The electronic nose represents a rather suitable instrumentation for the situation at hand, as it allows one to measure skin emissions daily from bed ridden persons in order to follow changes in the emission patterns observed from compresses or even other types of fabrics collected from proper places of the patient.

**Figure 4:**
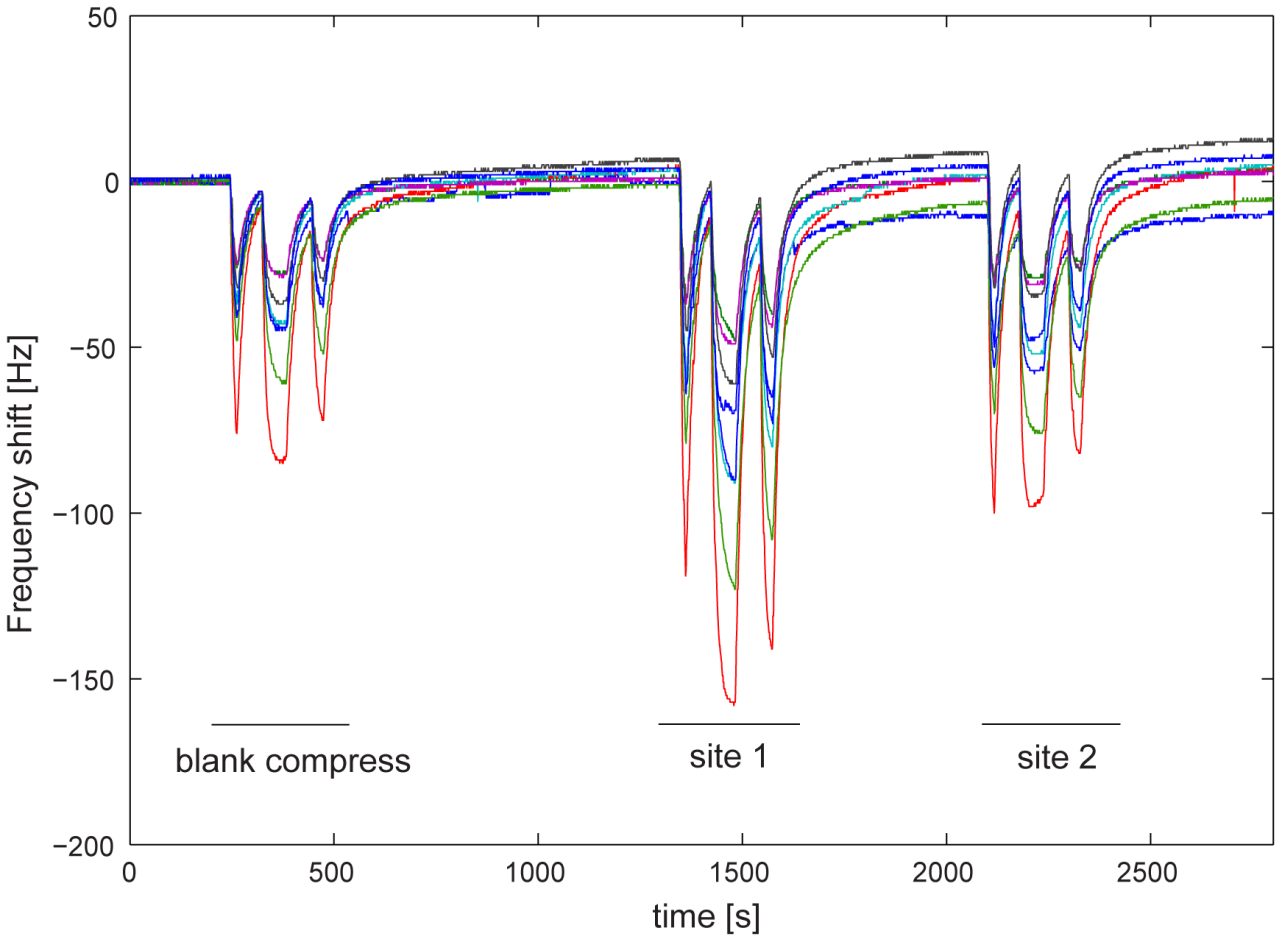
Signals from the eight sensors of the electronic nose. For each subject a set of three measurements is collected: blank compress, site 2 (uncompressed tissue) and site 1 (compressed tissue). Each measurement consists of three short exposures to the compress headspace.

**Figure 5:**
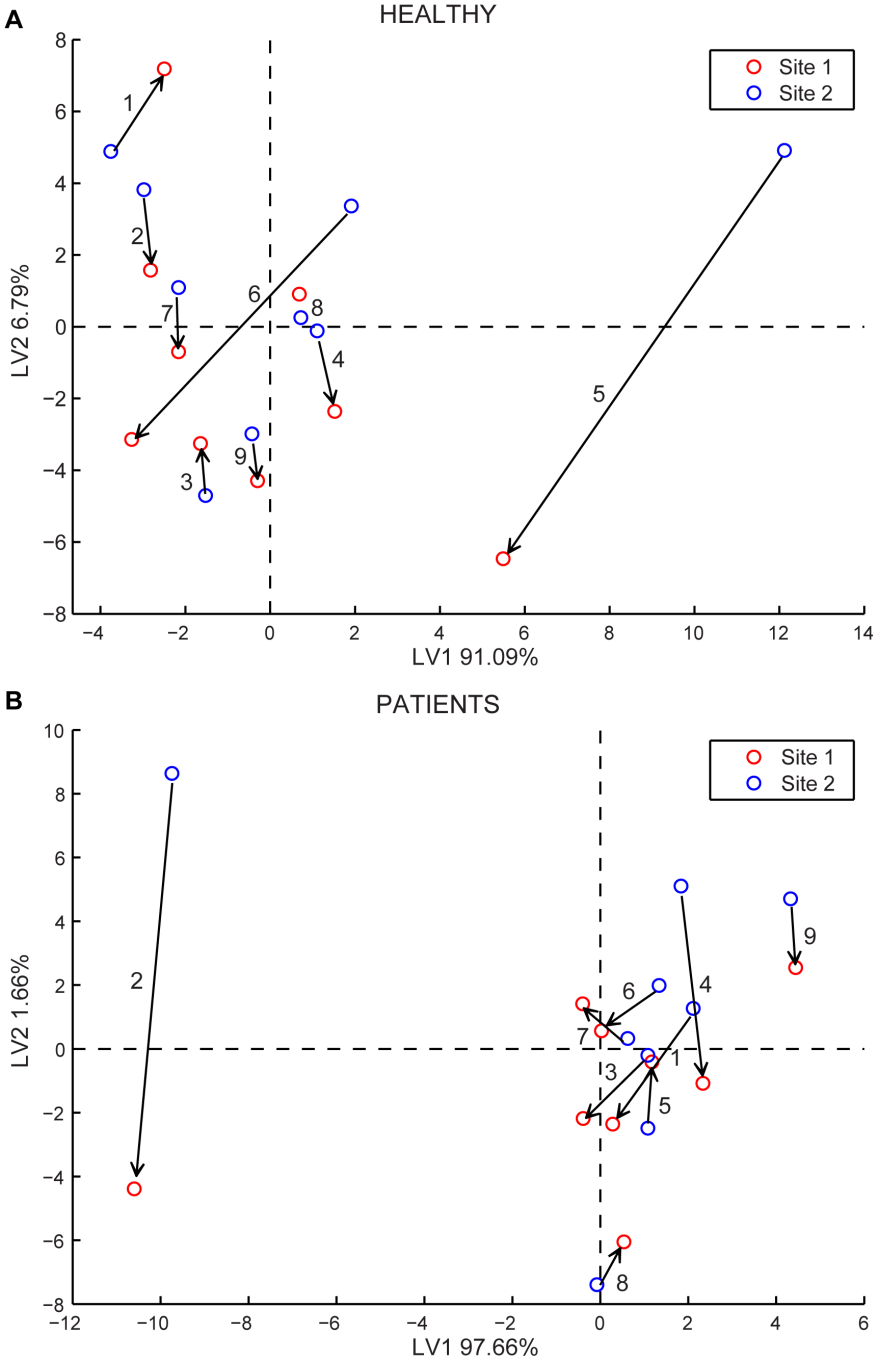
Score plots of the electronic nose data for healthy subjects and patients. The PLS-DA was calculated using the frequency shifts of the electronic nose for healthy (A) and patients (B). Red points label compressed site and blue points the non-compressed site. The arrows connect the points for the two sites for a given person, and indicate the direction of change from uncompressed to compressed tissue sites.

An example of raw data acquired from the electronic nose is shown in Figure 4. For each sample measurement, a set of three frequency shifts was gathered. Data were classified with Partial Least Square-Discriminant Analysis (PLS-DA) after the responses from the blank compress had been subtracted from those obtained for the compressed and uncompressed site, respectively. All calculations were performed in a Matlab programming environment. The electronic nose can discriminate between healthy persons and patients with a classification rate of about 83%. The PLS-DA model allows also for the discrimination between compressed and uncompressed sites within the two groups of healthy and patients (Figure 5). The results are similar to those obtained with GC-MS, as in the scores plot there is a trend for a decrease in the second component from the uncompressed to the compressed site. However, a major difference is that in this case the first latent variable explains almost the whole variance in the data (more than 90%). This suggests that the sensor array used in the electronic nose reacts mainly to the total amount of the emissions, rather than to its composition. For this reason, the differences in patient 1 that, as discussed for GC-MS data, are due to few peaks amongst the smallest ones, are not revealed by the PLS-DA model. A development of the electronic nose instrumentation requires therefore a deeper knowledge of the most relevant volatiles related to the emissions from compressed tissue to facilitate the choice of sensing materials oriented to “emission markers”.

### Long term goals

Our objective is to design a new non-invasive method for the early discovery of tissue damage as a complement to existing methods for the examination and characterization of pressure ulcers [23]. Further work will include measurements of skin emissions from the start of hospitalization of a group of patients using both GC-MS, an electronic nose and compresses or clothes with spots of color indicators for skin emissions. Changes in the emission pattern from a compressed tissue site, manifested as a continuous displacement of the point for a given patient in a principal component diagram, could be an indication of possible early tissue damage, which could be further investigated by other clinical techniques. Continuous movements of a point in a principal component diagram (constructed from electronic nose or -tongue measurements) have been used in several other situations, e.g. to identify bacterial infections of a cell culture in a fermenter [24]. A final goal would be to have color indicators, e.g. metallo-porphyrins, embedded directly in the compresses or in clothes worn by the patients.

## Conclusions

Our study is the first attempt to elucidate the difference in skin emissions from compressed and non-compressed tissue. We have demonstrated that the emissions from compressed tissue differ from those of non-compressed tissue. It was furthermore observed that there is a general difference in the emissions from hospitalized (bed-ridden and medicated) patients and healthy (non-hospitalized) persons, irrespective of the condition of the tissue. This difference is most probably due to the difference in medication and environment of the two groups. Within the two groups, however, there was a significant difference between compressed and non-compressed sites. We suggest that the early onset of tissue damage due to mechanical load can be detected as a gradual change in the emission pattern for a given person. It is also of interest to investigate further the importance of (one of) the peaks found for the patient obtaining pressure ulcer shortly after our study. Our results will hopefully point out a direction for the identification of volatile molecules on the skin for the early warning of tissue damage.
